# Li_2_MnO_3_ domain size and current rate dependence on the electrochemical properties of 0.5Li_2_MnO_3_·0.5LiCoO_2_ cathode material

**DOI:** 10.1038/s41598-017-13740-2

**Published:** 2017-10-16

**Authors:** Songyoot Kaewmala, Patcharapohn Chantrasuwan, Narinthron Wiriya, Sutham Srilomsak, Wanwisa Limphirat, Pimpa Limthongkul, Nonglak Meethong

**Affiliations:** 1 Materials Science and Nanotechnology Program, Department of Physics, Faculty of Science, Khon Kaen University, Muang, Khon Kaen 40002 Thailand; 2 Synchrotron Light Research Institute, Muang, Nakhon Ratchasima 30000 Thailand; 3 National Metal and Materials Technology Center, National Science and Technology Development Agency, Klong Luang, Pathumthani 12120 Thailand; 4 Nanotec-KKU Center of Excellence on Advanced Nanomaterials for Energy Production and Storage, Muang, Khon Kaen, 40002 Thailand; 5 Integrated Nanotechnology Research Center (INRC), Faculty of Science, Khon Kaen University, Muang, Khon Kaen, 40002 Thailand

## Abstract

Layered-layered composite oxides of the form xLi_2_MnO_3_·(1−x) Li*M*O_2_ (*M* = Mn, Co, Ni) have received much attention as candidate cathode materials for lithium ion batteries due to their high specific capacity (>250mAh/g) and wide operating voltage range of 2.0–4.8 V. However, the cathode materials of this class generally exhibit large capacity fade upon cycling and poor rate performance caused by structural transformations. Since electrochemical properties of the cathode materials are strongly dependent on their structural characteristics, the roles of these components in 0.5Li_2_MnO_3_·0.5LiCoO_2_ cathode material was the focus of this work. In this work, the influences of Li_2_MnO_3_ domain size and current rate on electrochemical properties of 0.5Li_2_MnO_3_·0.5LiCoO_2_ cathodes were studied. Experimental results obtained showed that a large domain size provided higher cycling stability. Furthermore, fast cycling rate was also found to help reduce possible structural changes from layered structure to spinel structure that takes place in continuous cycling.

## Introduction

Currently, lithium ion batteries have been widely used as energy storage devices in many applications, ranging from portable electronics to electric vehicles, due to their high-energy density and lightweight. The electrochemical properties of these batteries are largely determined by electrodes both anode and cathode materials. Layered oxide LiCoO_2_ is one of the common commercial cathode material used in lithium ion batteries. It exhibits a low practical capacity of about 140 mAh/g and poor cycling stability owing to its structural instability during cycling. Layered-layered cathode materials in the class of xLi_2_MnO_3_·(1−x) Li*M*O_2_ (*M* = Mn, Co, Ni) have been considered as an alternative cathode material for high energy density lithium ion batteries. They could potentially provide a high specific capacity of about 250 mAh/g with the operational voltage above 4.5 V higher than the current commercial LiCoO_2_ cathode (∼140 mAh/g)^1–5^. Layered-layered cathode materials also exhibit good cycling stability due to integration of a Li_2_MnO_3_ component which acts as the structural stabilizer ^6–8^. These materials are usually considered composite based cathodes because they often exhibit phase separation of the Li_2_MnO_3_ and LiCoO_2_ components. The particles of these materials usually consist of Li_2_MnO_3_ domains in a LiCoO_2_ matrix^9–12^. The Li_2_MnO_3_ component is electrochemically inactive and acts as a structural stabilizing phase in the cathode. However, these materials present high capacity fade up on repeated cycling, resulting from a phase transformation from a layered structure to a spinel-like structure. Phase transformation of the Li_2_MnO_3_ component during cycling brings about poor cycling stability and low rate performance^13–16^. This indicates that the electrochemical properties of these materials are dramatically dependent on the Li_2_MnO_3_ component^5,14,17,18^. The structural characteristic (i. e., cation ordering and phase separation) and morphology of the materials depend on their synthesis methods and conditions^19–24^. To solve these problems, there are numerous research studies that investigated the roles of the Li_2_MnO_3_ component on the electrochemical performance of layered-layered composite materials. Bareño *et al* ^25^. studied 0.5Li_2_MnO_3_·0.5LiCoO_2_ using a combination of techniques, including SEM, XRD, and XAS. They observed that the materials are nanocomposites consisting of Li_2_MnO_3_ and LiCoO_2_ domains. Croy *et al* ^18^. found that the voltage drop phenomenon related to capacity decay upon cycling was increased with increasing Li_2_MnO_3_ content due to structural changes during cycling. Ghanty *et al* ^26^. showed particle size of a xLi_2_MnO_3_·(1−x) LiMn_0.375_Ni_0.375_Co_0.25_O_2_ material and domain size of Li_2_MnO_3_ within the LiMn_0.375_Ni_0.375_Co_0.25_O_2_ matrix depending on Li_2_MnO_3_ content. As the Li_2_MnO_3_ content increases, both particle size and the Li_2_MnO_3_ domain size increase. The cycling stability was found to increase with Li_2_MnO_3_ content owing to the phase transformation of the Li_2_MnO_3_ component which might be effectively retarded by a larger Li_2_MnO_3_ domain size. However, the impacts of Li_2_MnO_3_ domain size on cycling stability and the phase transition during cycling is still not well understood.

The aim of this work is to investigate the influence of the Li_2_MnO_3_ domain size and current rates on the electrochemical properties of 0.5Li_2_MnO_3_·0.5LiCoO_2_. The Li_2_MnO_3_ domain size can be changed using different preparation methods such as sol-gel and ball-milling methods. We present that the electrochemical properties of the cathode material can be controlled by their Li_2_MnO_3_ domain size and testing conditions.

## Results and Discussion

**Figure 1:**
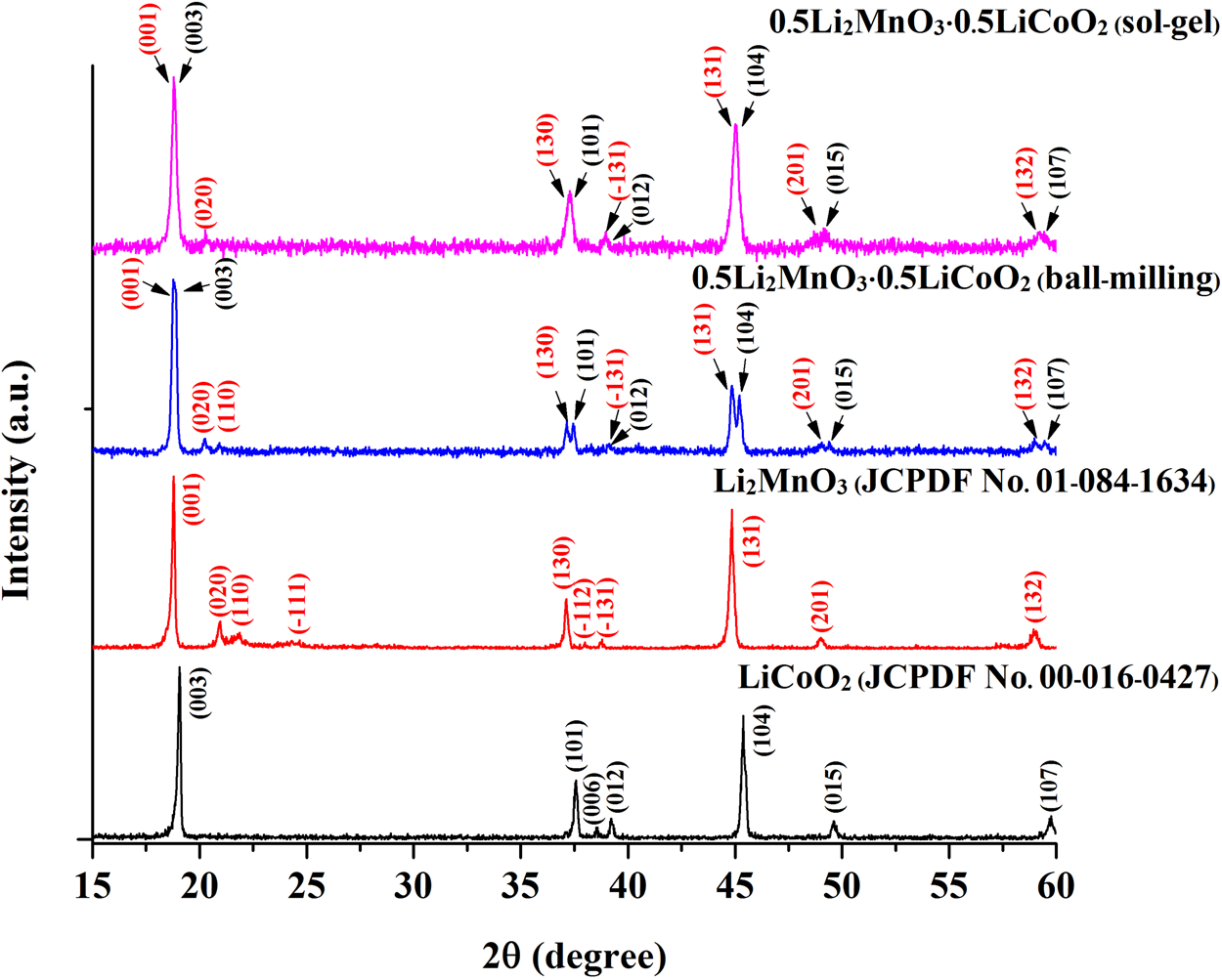
X-ray diffraction patterns of the LiCoO_2_, Li_2_MnO_3_, and 0.5Li_2_MnO_3_·0.5LiCoO_2_ materials prepared using sol-gel and ball-milling methods.

**Figure 2:**
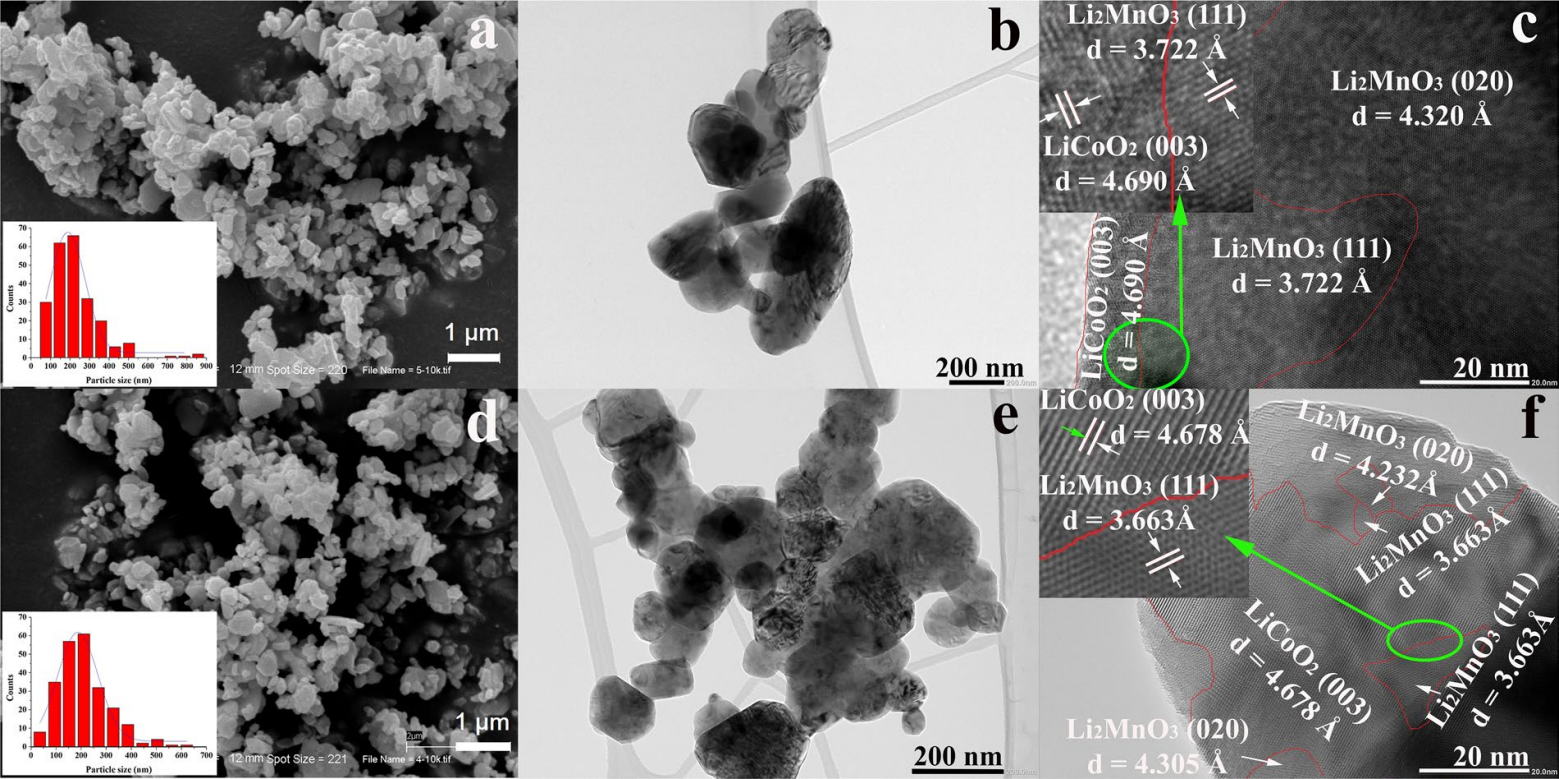
SEM, TEM and HRTEM images of the 0.5Li_2_MnO_3_·0.5LiCoO_2_ samples prepared using ball-milling (**a**,**b**,**c**) and sol-gel (**d**,**e**,**f**) methods, respectively.

The crystal structure characterization of the prepared samples was determined using an XRD method and the results given in Fig. 1. XRD patterns of the LiCoO_2_ and Li_2_MnO_3_ powders synthesized using the sol-gel method could be indexed with a rhombohedral crystal system (space group $R\overline{3}m$) and a monoclinic crystal system (space group *C*2/*m*), respectively. The XRD peaks of the 0.5Li_2_MnO_3_·0.5LiCoO_2_ sample prepared by a ball-milling method were clearly separated especially at a 2θ of about 35° and 45°.The separated peaks could be identified as the diffraction patterns of LiCoO_2_ and Li_2_MnO_3_ phases. This indicates that the ball-milling method provided a large phase separation between LiCoO_2_ and Li_2_MnO_3_ components. Conversely, the XRD pattern of the 0.5Li_2_MnO_3_·0.5LiCoO_2_ sample prepared by a sol-gel method showed quite broad and overlapped peaks owing to the sol-gel method offers phase separation of LiCoO_2_ and Li_2_MnO_3_ components of about 5 to 10 nm length scale as demonstrated in Fig. 2(c), which is smaller than the phase separation that was observed in the ball-milled sample. The separated XRD peaks and broad XRD peaks occurrences confirmed the existence of phase separation, and that these materials are a composite (and not solid solution). The phase separation behavior can be confirmed by the TEM results illustrated in Fig. 2(c),(f). Additionally, the weak peaks between 20 and 22° 2θ were still found in both sol-gel and ball-milled samples resulting from the ordering of cations in the transition metal layer of Li_2_MnO_3_ in Li_2_MnO_3_-like regions^27–29^.

The morphology of the composite samples was examined using SEM and TEM as shown in Fig. 2. The prepared materials obtained from the ball-milling and sol-gel methods have similar average particle sizes of 240 nm and 250 nm, respectively. TEM images reveal quite similar particle size distributions as well, which are quite broad showing particles with sizes ranging from a few tens of nm to more than 400 nm. In order to make a reasonable comparison of the Li_2_MnO_3_ domain size of the two composite samples, several individual particles with the particle size of around 200 nm, which is similar to the average particle size of both samples, were selected to examine using HRTEM as presented in Fig. 2(c),(f). The results show that the Li_2_MnO_3_ (space group *C*2/*m*) and LiCoO_2_ (space group $R\overline{3}m$) regions could be clearly observed. The presence of the Li_2_MnO_3_ and LiCoO_2_ domains confirmed that these materials formed a composite system consistent with the XRD results, corresponding to previous reports^10,19,30–32^. Furthermore, the ball-milled sample had Li_2_MnO_3_ domain sizes of about 40–60 nm, while the domain size in sol-gel sample was smaller, ranging from 5–20 nm. The different Li_2_MnO_3_ domain sizes in the 0.5Li_2_MnO_3_·0.5LiCoO_2_ cathodes obtained from different synthesis methods may have a crucial effect on their electrochemical properties such as Li_2_MnO_3_ phase activation, affecting the phase transformation from a layered structure into a spinel structure upon cycling.

**Figure 3:**
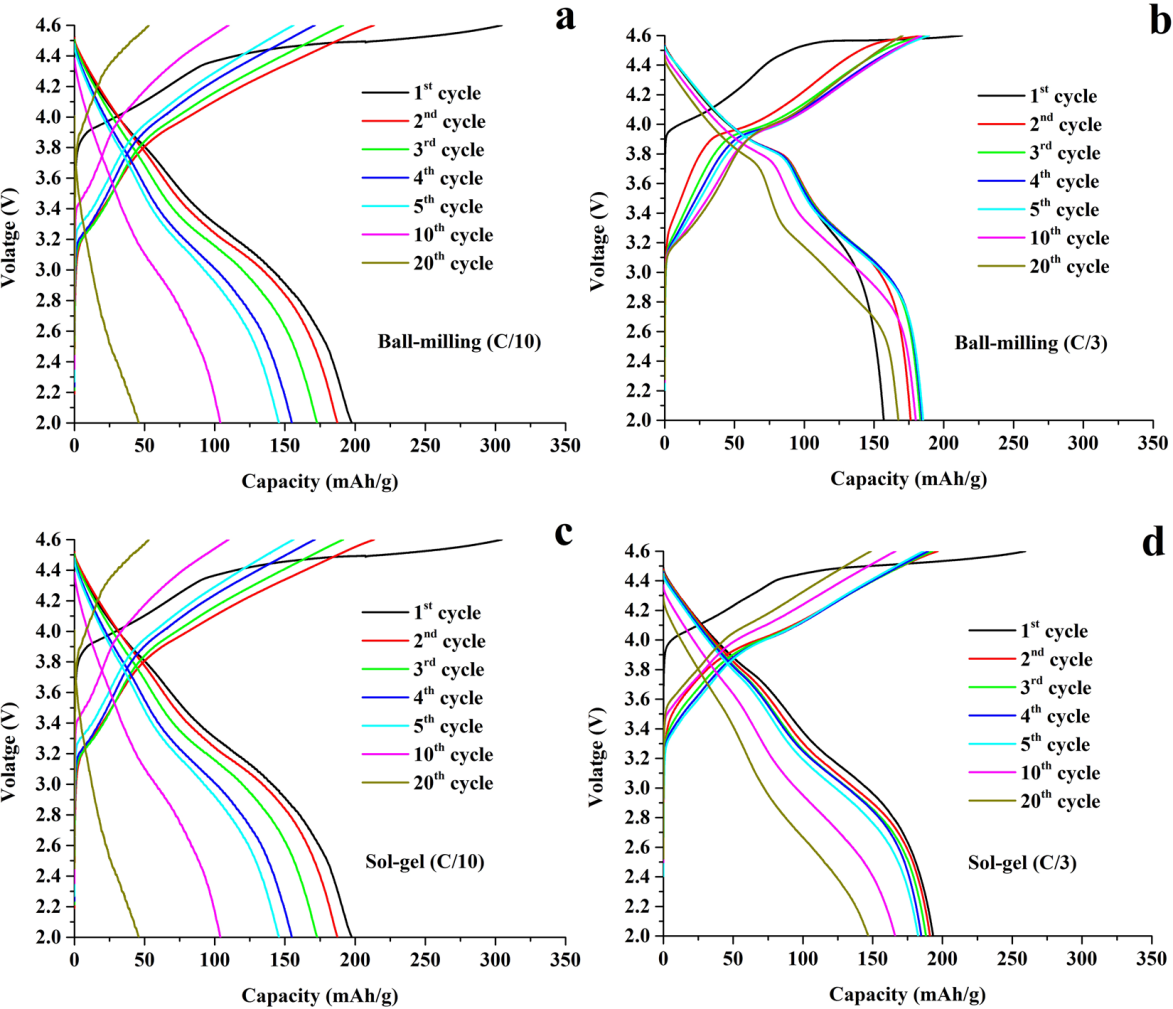
Charge-discharge profiles of the 0.5Li_2_MnO_3_·0.5LiCoO_2_ samples prepared by ball-milling (**a** and **b**) and sol-gel (**c** and **d**) methods cycled at C/10 and C/3 rates.

The 0.5Li_2_MnO_3_·0.5LiCoO_2_ cathode materials prepared by the sol-gel and ball-milling methods were cycled between 2.0–4.6 V at C/10 and C/3 as shown in Fig. 3. The first charging profiles of both samples showed two voltage plateaus at ∼3.9 and ∼4.5 V that were clearly observed in the initial charge. The first voltage plateau at ∼3.9 V occurred due to the oxidation of Co^3+^ to Co^4+^ in the LiCoO_2_ component. During this reaction, depletion of lithium ions from the lithium layer was compensated by lithium ions, diffusing from an octahedral site in the manganese layer of the Li_2_MnO_3_ component to tetrahedral sites in the lithium depleted layer. This makes the overall structure of the composite materials becomes more stable during cycling^6,33,34^. The second voltage plateau corresponds to the extraction of lithium and oxygen from the Li_2_MnO_3_ component to form Li_2_O and electrochemically active MnO_2_ phases. Li_2_O evolution brings about an increase in the first charge capacity and a large initial irreversible capacity^6,7,19^. During the initial discharging cycles, lithium ions intercalation into the MnO_2_ component resulting in the reduction of Mn^4+^ to Mn^3+^ until the LiMnO_2_ phase was completely formed. The layered LiMnO_2_ phase is usually transformed into a spinel-like phase upon cycling leading to capacity and voltage decay as the cycle number increases. The ability to control the phase transition of the layered Li_2_MnO_3_ phase into the electrochemically active MnO_2_ component is a crucial key to improve the cycling stability of layered-layered composite materials. The sloping discharge profiles at ∼3.8 to 2 V correspond to lithium ion interactions in the layered Li_x_MO_2_ species^35^. The distinct voltage plateau at ∼3.9 V corresponding to a lithium insertion into the LiCoO_2_ component can clearly be seen for the ball-milled samples as demonstrated in Fig. 3(a) ^36^, because the ball-milling method provides a larger phase separation between the LiCoO_2_ and the Li_2_MnO_3_ components. This result is consistent with the XRD experimental results. As shown previously, voltage fade was observed in all of the electrodes in this study. However, the voltage fade resulting from phase transformation from the layered structure to the cubic spinel structure during cycling is a challenging problem that inhibits practical uses of the layered-layered oxide composite cathodes^20,37,38^. Therefore, studying governing parameters that can reduce this voltage fade is crucial. As can be seen, sol-gel samples exhibited larger voltage decay than the ball-milled sample due to the larger Li_2_MnO_3_ domain size that retarded efficient activation of Li_2_MnO_3_ component since the large Li_2_MnO_3_ domain size is difficult to activate. Moreover, the samples cycled at a high current rate (C/3) and provided a lower voltage drop than the sample cycled at a low current rate (C/10). Since high current cycling (C/3) can reduce the extraction of lithium and oxygen from the Li_2_MnO_3_ component, the LiMnO_2_ component evolution is retarded and most of Li_2_MnO_3_ phase remains during extended cycles. The existence of a Li_2_MnO_3_ component in a continuous cycle indicates that it acts as a structural stabilizer for the cathode during cycling enabling high cycling stability^14,26^.

**Figure 4:**
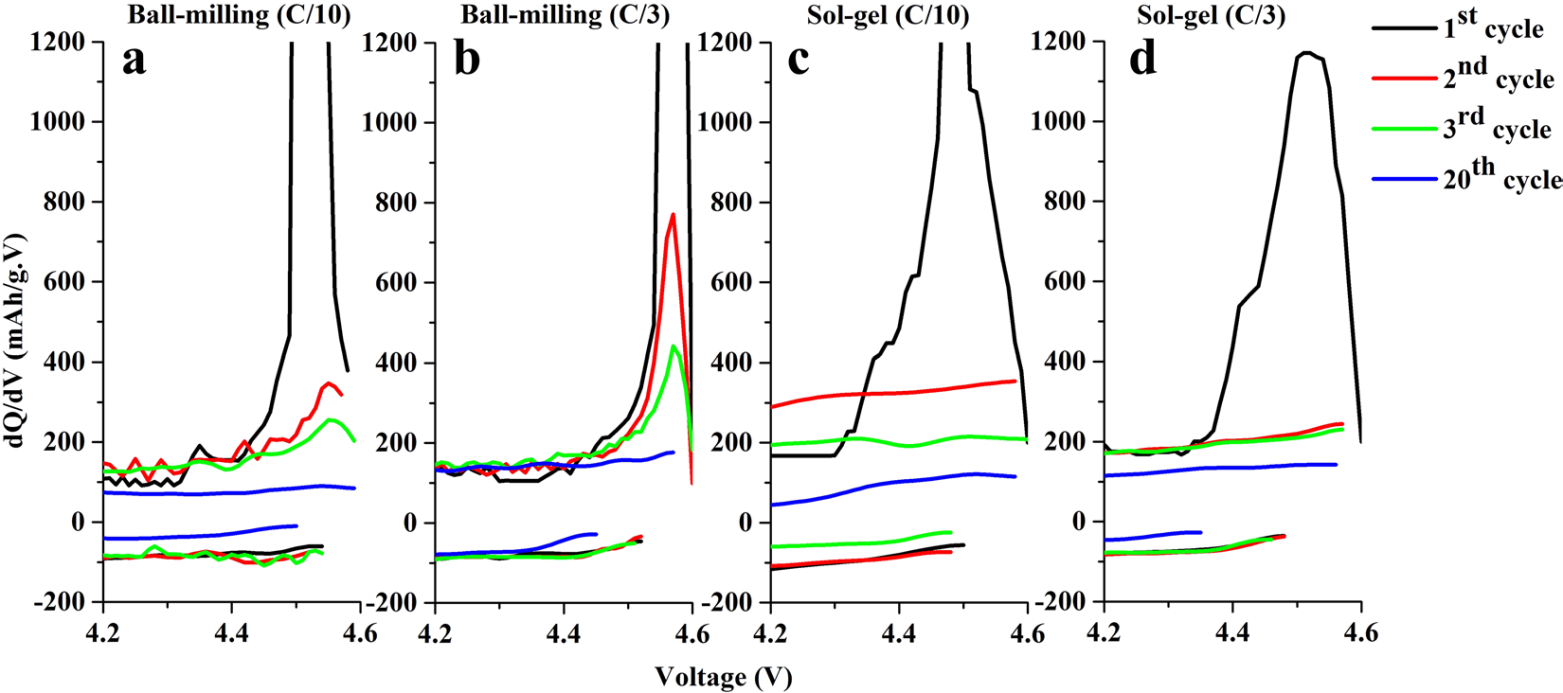
Differential capacity plots of the 0.5Li_2_MnO_3_·0.5LiCoO_2_ samples prepared by ball-milling (**a**,**b**) and sol-gel (**c**,**d**) methods cycled at C/10 and C/3 rates.

Differential capacity plots obtained from the differentiation of capacity as a function of voltage profiles for both samples at different cycle numbers and current density at selected voltages ranging between 4.2 to 4.6 V are showed Fig. 4. The peak during the oxidation reaction at about 4.5 V was due to lithium and oxygen extraction from the Li_2_MnO_3_ component^14,26^. In the sol-gel samples cycled at C/10 and C/3, the oxidation peaks were only observed in the first cycle, but the broad oxidation peaks around 4.35–4.40 are presented in the next cycles corresponding to oxidation reaction of Co^4+^ to Co^3+^ in LiCoO_2_ component^39^. The LiCoO_2_ activation becomes notable at the next cycles, due to the Li_2_MnO_3_ component was completely activated at the first charge in the electrodes prepared by sol-gel method that had a small Li_2_MnO_3_ domain size, which is easily activated at the initial charge. In the ball-milled samples cycled at C/10 and C/3, the oxidation peaks at approximately 4.5 V could still be clearly seen in subsequent cycles, since activation of the Li_2_MnO_3_ component was incomplete at the first charge. The ball-mill method provided a larger Li_2_MnO_3_ domain size leading to difficulties in lithium and oxygen extraction from the Li_2_MnO_3_ component. The presence of an oxidation peak indicates that Li_2_MnO_3_ activation can still occur and it confirmed that the Li_2_MnO_3_ component remained during the subsequent cycles. The remaining Li_2_MnO_3_ component at extended cycles results in the ability to stabilize the structure leading to high cycling stability. Furthermore, the ball-milled cathode cycled at high current rate (C/3) resulted in higher intensity of residual oxidation peaks at 4.5 V than the ball-milled cathode cycled at slower current rate (C/10). This is because Li_2_MnO_3_ activation can effectively be reduced by a high current rate cycling, which is more effective than a slower current rate cycling. Because the cycling at a high current rate, lithium and oxygen ions are difficultly extracted from Li_2_MnO_3_ structure to form LiMnO_2_ phase, resulting in a small amount of the LiMnO_2_ phase that can transform to the spinel-like phase. This causes the electrodes cycled at high current rate (C/3) to have a higher cyclability than the electrodes cycled at a slower current rate (C/10).

**Figure 5:**
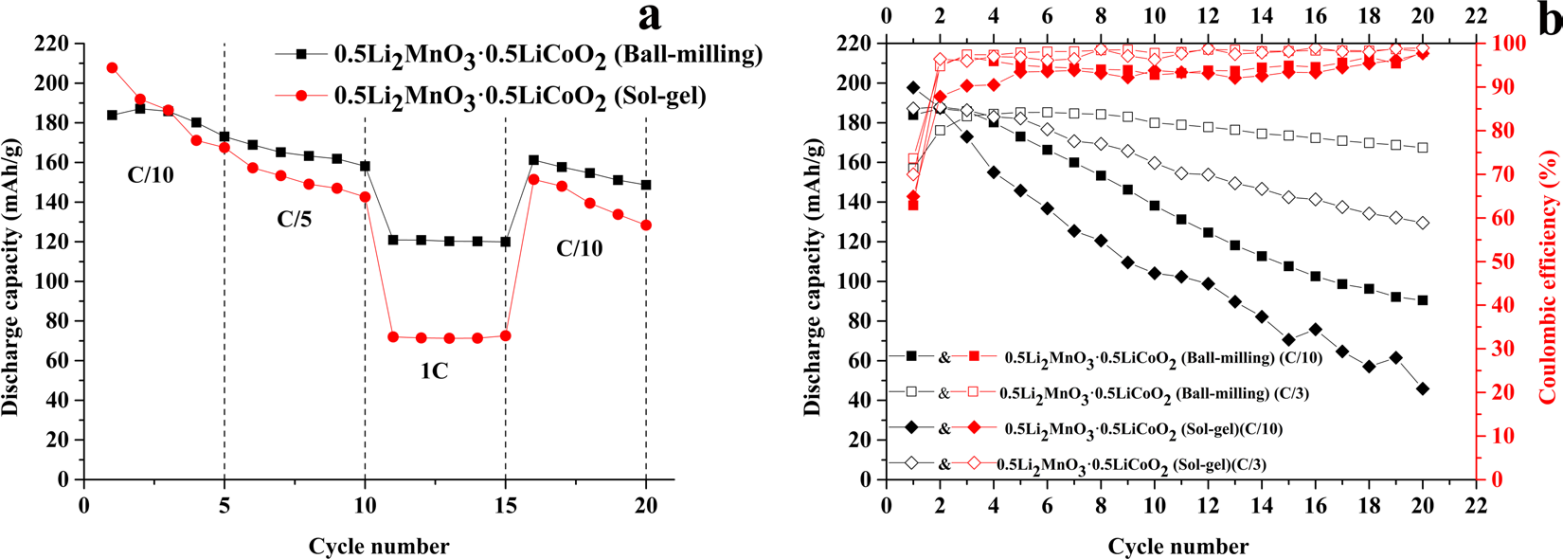
Rate capability performances (**a**), cycling stabilities, and coulombic efficiencies cycled at C/10 and C/3 rates (**b**) of the 0.5Li_2_MnO_3_·0.5LiCoO_2_ samples prepared by sol-gel and ball-milling methods.

Rate capability of the 0.5Li_2_MnO_3_·0.5LiCoO_2_ materials prepared using the ball-milling method and the sol-gel method is shown in Fig. 5(a). The ball-milled sample exhibits a higher rate capability than the sol-gel sample due to the larger Li_2_MnO_3_ domain size in the ball-milled sample, which can retard the layered structure transformation into the defective spinel-like structure. However, both cathode materials revealed low rate performance. This occurred since these electrodes were cycled at a slow C/10 rate for their first 5 cycles, leading to almost a complete activation of the Li_2_MnO_3_ component. As demonstrated in Fig. 5(b), the initial discharge capacities of the sol-gel cathodes were higher than the ball-milled cathodes. This results from the sol-gel cathodes providing a smaller Li_2_MnO_3_ domain size. Increasing Li_2_MnO_3_ activation can take place in the first cycle leading to a higher initial capacity. During the first four cycles, the discharge capacities of both sol-gel and ball-milled cathodes cycled at high current rate were low and increased gradually for the first few cycles. Cycling at a high current rate can retard Li_2_MnO_3_ phase activation in the first cycle, and this activation can occur in subsequent cycles until the Li_2_MnO_3_ phase is completely activated, resulting the higher capacities during the first few initial cycles. The capacities of ball-milled cathodes gradually increased for the first few cycles, which can be observed from the electrodes cycled by both high and low current rates, because a larger Li_2_MnO_3_ domain size was obtained by the ball-milling method. Although the electrode was cycled at a low current rate (C/10), the Li_2_MnO_3_ phase activation during the first cycle was still not completed. After that, the capacities decreased at different rates depending on current rates. The electrode cycled at the lower current rate exhibited a larger capacity decay than the one cycled at a high rate. A high current rate can effectively reduce the phase transformation of the Li_2_MnO_3_ component during cycling bringing about high cycling stability. Furthermore, the ball-milled cathodes exhibited higher cycling stability and rate capability than sol-gel cathodes due to the larger Li_2_MnO_3_ domain size obtained from the ball-milled method, potentially reducing the phase transition from then layered structure to the spinel structure during cycling. These results confirm that the mitigation of phase transformation of the Li_2_MnO_3_ component was largely controlled by the Li_2_MnO_3_ domain size and testing conditions (current rate). A larger Li_2_MnO_3_ domain size and suitable current rate cycling can efficiently retard the spinel phase evolution upon cycling. Coulombic efficiencies of the 0.5Li_2_MnO_3_·0.5LiCoO_2_ materials prepared using the ball-milling and sol-gel methods cycled at C/3 and C/10 current rates are shown in Fig. 5(b). As can be seen, these cathodes provide low coulombic efficiencies during first few cycles due to irreversible formation of the Li_2_O phase after the Li_2_MnO_3_ component activation. A recent work^40^ has shown that a core-shell structured nanocomposite of FePO_4_ and Li_2_MnO_3_ can eliminate the large irreversible capacity of the Li-rich materials. The FePO_4_ on the surface of Li_2_MnO_3_ can also serve as a host for Li ions that were deintercalated from Li_2_MnO_3_ during the initial charging process and the initial coulombic efficiency can be 100%. For the electrodes cycled using a slow rate (C/10), the sol-gel electrode exhibits a lower coulombic efficiency than the ball-milled electrode because the sol-gel method provides a smaller Li_2_MnO_3_ domain size. The smaller Li_2_MnO_3_ domain size can easily be activated to form large amounts of the Li_2_O and MnO_2_ phases leading to higher irreversible capacity loss during the first few cycles. In addition, the electrodes cycled using a higher C-rate (C/3) show a higher coulombic efficiency than those cycled using a slower rate (C/10). This result also happens for the same reason. Cycling at a higher C-rate can mitigate the Li_2_MnO_3_ component transformation to the Li_2_O and MnO_2_ phases.

## Conclusions

In this work, we studied and found that the impacts of the Li_2_MnO_3_ domain size and current rate on the electrochemical properties of 0.5Li_2_MnO_3_·0.5LiCoO_2_ composite materials. The high cycling stability and rate performance of composite cathode materials are increased as Li_2_MnO_3_ domain size increases, which are suitable for high rate applications. The Li_2_MnO_3_ domain size within the LiCoO_2_ matrix can be varied using different preparation methods. A ball-milling method provided larger Li_2_MnO_3_ domains than a sol-gel method. The larger Li_2_MnO_3_ domain size and high current rate could retard phase transformation of the Li_2_MnO_3_ component to the spinel-like phase. Therefore, the Li_2_MnO_3_ component remaining in subsequent cycles is a crucial strategy for improving the cycling stability and rate performance of layered-layered composite-based cathode materials. However, the Li_2_MnO_3_ domain size also must be optimized to obtain higher performance layered-layered composite cathode materials for lithium ion batteries.

## Methods

### Cathode material preparation: Ball-milling method

Li_2_MnO_3_ compound was prepared by a sol-gel route using CH_3_COOLi·2H_2_O (Aldrich), and Mn(CH_3_COO)_2_∙4H_2_O (Aldrich) as the precursors, ascorbic acid as the chelating agent (ascorbic acid to metal ion molar ratio 0.5:1) and ethanol was used as the solvent. Stoichiometric amounts of the starting materials and ascorbic acid were dissolved in 200 mL of ethanol and stirred for approximately 3 hours at 80^ °^C. After the mixture formed a gel, the gel was calcined at 300^ °^C for 3 hours and then again at 800^ °^C for 16 hours in air. LiCoO_2_ compound was prepared by the sol-gel approach using CH_3_COOLi·2H_2_O (Aldrich), and Co(CH_3_COO)_2·_·4H_2_O (Aldrich) as the precursors and ascorbic acid as the chelating agent (ascorbic acid to metal ion molar ratio of 0.5:1) and ethanol was used as a solvent. Stoichiometric amounts of the starting materials and ascorbic acid were dissolved in 200 mL of ethanol and stirred for approximately 3 hours at 80^ °^C. After the mixture formed a dried gel, the dried gel was calcined at 800^ °^C for 10 hours in air. The 0.5Li_2_MnO_3_·0.5LiCoO_2_ was prepared by a ball-milling method. Stoichiometric amounts of Li_2_MnO_3_ and LiCoO_2_ (molar ratio of 1:1) were mixed in ethanol. After that, the mixed powder and 13 mm diameter alumina balls (ball to powder weight ratio of 20:1) were contained in a 120 mL Teflon bottle and then placed on a horizontal ball mill for 72 hours with a rotational speed of 180 rpm. The mixture was dried at 80^ °^C for 10 hours in a vacuum oven. The mixture was calcined at 800^ °^C for 10 hours in air and then furnace cooled to room temperature.

### Cathode material preparation: Sol-gel method

Another 0.5Li_2_MnO_3_·0.5LiCoO_2_ sample of cathode material was synthesized via a sol-gel method. CH_3_COOLi·2H_2_O (Aldrich), Mn(CH_3_COO)_2_∙4H_2_O (Aldrich), and Co(CH_3_COO)_2·_·4H_2_O (Aldrich) were used as the precursors and ascorbic acid as the chelating agent (ascorbic acid to metal ion molar ratio of 0.5:1). Stoichiometric amounts of the starting materials and ascorbic acid were dissolved in 200 mL of ethanol and stirred for approximately 3 hours at 80^ °^C. After the mixture became a dried gel, it was first calcined at 300^ °^C for 3 hours in air. The mixture was then ground with agate mortar and pestle, calcined again at 800^ °^C for 10 hours in air and then cooled to room temperature in the furnace.

### Structure and morphology characterization

X-ray diffraction (XRD) technique (X’pert Pro, PANalytical) was used to examine the crystal structure of the samples using Cu-Kα radiation at 40 kV and 30 Ma. The data were collected with a step size of 0.02° over a 2θ range from 15° to 80°. Morphology of the 0.5Li_2_MnO_3_·0.5LiCoO_2_ samples and the existence of Li_2_MnO_3_ and LiCoO_2_ domains were examined by scanning electron microscopy (SEM) (Zeiss, LEO-1450VP) and transmission electron microscopy (TEM) (JEOL, JEM-2100 Plus).

### Electrochemical measurements

The active materials were mixed with polyvinylidene fluoride (PVDF Kynar 2801, Arkema) in N-methyl-2-pyrollidone (NMP) (Aldrich) as a binder and carbon black (Alfa Aesar) with a weight ratio of 78:11:11. The mixture was blended in a horizontal shaker for 2 hours and used to coat a sheet of aluminum foil using a doctor blade. It was then dried in a vacuum oven at 80^ °^C for 10 hours. The electrodes were assembled by using a Swagelok cell-type in an Argon filled glovebox. A 0.75 mm thick Li metal was used as an anode. 1 M LiPF_6_ in EC: DMC: DEC = 4:3:3 by volume (MTI) was used as an electrolyte. Celgard 2400 was used as a separator. Galvanostatic cycling tests were performed using a multichannel tester (BST8-MA, MTI) with a cut-off voltage of 2.0–4.6 V at 30^ °^C.
